# Antibiotic Consumption Trends in China: Evidence From Six-Year Surveillance Sales Records in Shandong Province

**DOI:** 10.3389/fphar.2020.00491

**Published:** 2020-04-17

**Authors:** Yan Song, Zhiyan Han, Kuimeng Song, Tianmin Zhen

**Affiliations:** ^1^Shandong Institute of Medicine and Health Information, Shandong First Medical University & Shandong Academy of Medical Sciences, Jinan, China; ^2^School of Health Care Management, Shandong University, Jinan, China

**Keywords:** antibiotic consumption, antimicrobial resistance, antimicrobial stewardship, rational drug use, China

## Abstract

**Background:**

The overuse of antibiotics is a serious public health problem in China, causing a high rate of antimicrobial resistance. This study identified the trends of antibiotic consumption in China to provide evidence for further intervention.

**Method:**

The six-year surveillance data on antibiotic sales from 2012 to 2017, which served as a proxy for consumption, were collected from 39 public health care facilities in Shandong province, including three tertiary hospitals, six secondary hospitals, and 30 primary health centers. Based on the Anatomical Therapeutic Chemical (ATC)/DDD methodology, antibiotic consumption was formulated in defined daily doses (DDD) per 1,000 inhabitants per day (DID).

**Results:**

The total antibiotic consumption among all health care settings increased from 16.07 DID in 2012 to a peak of 17.44 DID in 2015 and then decreased to 11.35 DID in 2017 with a 34.90% reduction. J01C (beta-lactam antimicrobials, penicillin), the most frequently used antibiotic class, accounted for 36.32% of the total DID. Consumption of carbapenems increased from 0.029 DID in 2012 to 0.08 DID in 2017. Parenteral antibiotics accounted for nearly 40% of the total consumption. Compared with the 2012 figures, the 2017 consumption showed a small increase in hospital sector that was compensated by the decrease in community care.

**Conclusion:**

A substantial reduction in total antibiotic consumption was observed in China from 2012 to 2017. However, the extensive consumption of broad-spectrum antimicrobials, high proportion of parenteral antibiotic use, and increased use of last-resort antibiotics attracted public health concerns.

## Introduction

Antimicrobial resistance (AMR) driven by antibiotic consumption is a growing threat to global public health (Song, 2018; Zhang et al., 2019). China is among the world’s largest producers and consumers of antibiotics, which are widely used for disease treatment in humans and livestock, as well as prophylaxis and growth promoters for the latter (Qiao et al., 2018). China was estimated to be the second largest consumer of antibiotics in the world in 2010 in terms of the volume of antibiotics sold for human use in retail and hospital pharmacies (Van Boeckel et al., 2014).Antibiotics accounted for nearly 20% of total drug sales in health care facilities (Xiao et al., 2013). Moreover, the percentage of prescriptions that include an antibiotic in China was 41%-60%, which is above the recommended threshold of 30% by the World Health Organization (WHO) (Yin et al., 2013; Wang et al., 2014; Ren et al., 2016). A survey conducted by the Chinese Ministry of Health in 2011 showed that the annual use of antibiotics per capita reached 138 g, which was 10 times more than that of the United States (China News, 2015). In addition, a body of literature revealed that a large proportion of patients received combined therapy with multiple antibiotics (Wang and Chou, 2013). Many reasons can be attributed to the high consumption of antibiotics in China. The main factors affecting misuse of antibiotics lie in physicians’ lack of sufficient knowledge about appropriate use and pressure from patients who believe that antibiotics can quickly alleviate the symptoms of a disease (Xiao et al., 2013). Another widely believed influence factor is the provision of financial compensation for drug sales to medical institutions (Song et al., 2014b). In China, public hospitals can charge 15% on top of wholesale price of medicines. This drug mark-up was originally designed to compensate health care institutions that provide services at below-cost prices. However, this approach can easily result in serious health hazards as physicians tend to over-prescribe unnecessary medicines, including antibiotics.

The increase in AMR triggered a surge of interventions on antibiotic use in China, especially after the implementation of a new health system reform in 2009 (Wang et al., 2016; Tang et al., 2018). In this latest round of health system reform, the prescription authority for antibiotics in primary health care facilities was limited to drugs listed in the Essential Medicines List (EML); these medicines should be sold at zero mark-up to eliminate the benefit chain between facilities, doctors, and medicines (Song et al., 2014a). At present, the zero-markup policy has been extended to all public hospitals. On July 1, 2011, the Chinese Ministry of Health launched a three-year nationwide campaign to control the unreasonable use of antibiotics, mainly targeting public secondary and tertiary hospitals (Chinese Ministry of Health, 2011). In 2012, the Chinese government issued the *Administrative Regulations on the Clinical Application of Antimicrobial Agents*, which is regarded as the strictest control on antibiotic prescription to date (Xiao and Li, 2013). Antibiotics are categorised into three groups: nonrestricted, restricted, and controlled. Controlled antibiotics are excluded from the EML for primary care. Prescriptions of restricted or controlled antibiotics are subject to strict administrative restrictions. Penalties are applied for violating the rules (Chinese Ministry of Health, 2012). A recently released five-year national action plan aims to fight against AMR and ensure that antibiotics become prescription-only products in retail pharmacies by 2020 (Chinese Ministry of Health, 2016).

Reliable evidence shows the need to reflect on the effects of these policies. Previous studies have focused more on the changes of the number of antibiotic prescriptions and their cost (Liang et al., 2014; Song et al., 2014b; Liu et al., 2015). Few studies have been designed sufficiently to examine the changes in antibiotic consumption based on ATC/DDD methodology, which could demonstrate the epidemiology of antibiotic resistance with a higher resolution (Wushouer et al., 2017; Qu et al., 2018). Moreover, these findings have been mostly limited to tertiary hospitals and thus cannot reflect adequately reflect the actual situation of antibiotic use in China (Wushouer et al., 2017; Qu et al., 2018). Consequently, there is an urgent need for more surveillance data of antibiotic consumption covering various types of health care institutions at different levels and in various regions.

Therefore, this study aimed to provide the latest evidence on antibiotic consumption in China by identifying the trends and patterns based on a six-year surveillance in Shandong province. The findings of this study would assist in monitoring the increase in drug resistance and developing appropriate use interventions, as well as provide baseline information for future assessment and regional comparison.

## Method

### Setting and Data Collection

The surveillance data on antibiotic sales from 39 public health care facilities in Shandong province from 2012 to 2017 were analyzed retrospectively to document the trends in antibiotic consumption in China.

Shandong province is among China’s densely populated and economically developed regions. In 2017, its population was 100.06 million, ranking second among 31 provinces in China; its gross domestic product (GDP) in the same year was CNY 7267.82 billion (Shandong Provincial Bureau of Statistics, 2018). About 4.92% of the GDP in Shandong was spent on health. In 2017, Shandong had 2.65 physicians, 2.93 nurses, and 5.85 beds per 1,000 residents in the health care sector. The share of public health care facilities in this region in terms of patient visit was over 85%. In 2017, the numbers of patients treated in hospitals and primary health care facilities (PHCs) were 225.18 million (34.94%) and 393.08 million (60.99%), respectively. Among these patients, 190.68 million (84.68%) patients were treated in public hospitals (Shandong Health and Family Planning Commission, 2018). In July 2011, all public PHCs in Shandong adopted the EML and drug zero mark-up policy. The zero mark-up policy was fully implemented in county-level public hospitals in 2015 and then secondary and tertiary public hospitals in urban areas in mid-2016.

The 39 public health care facilities in this study were sampled hierarchically from three cities based on geographical and socio-economic factors. The breakdown was three tertiary hospitals, six secondary hospitals, and 30 PHCs. Data were derived from the drug information management system at each facility. The collected information contained generic name, unit strength, pack size, quantity of packs, route of administration, price, amount of sales, and manufacturer information.

### Data Analysis

Microsoft Excel 2010 was used for data management and analysis. Sales data were coded in accordance with Anatomical Therapeutic and Chemical (ATC) classification and measured by defined daily dose (DDD) based on recommendations of the WHO Collaborating Center for Drug Statistic Methodology (WHO, 2013). Records of “J01” (antibacterial for systemic use) from 2012 to 2017 were extracted for analysis.

In the ATC classification system, drugs are classified in groups at five different levels. Within the ATC subgroup J01, 98 unique chemical substance names were identified in single or combination antibiotics. They were first aggregated into 24 ATC-4 classes and then into 9 ATC-3 groups. We calculated the DDDs for systemic antibiotics (J01) and its subsequent subgroups, such as penicillin (J01C) and penicillin with extended spectrum (J01CA). A full list of subgroups is displayed in **Table 1**. These antibiotics were grouped further according to administration route (oral vs. injection) and types of health care institutions (community sectors, secondary hospitals, and tertiary hospitals).

**Table 1:** Antibiotic consumption by ATC-4 classifications in Shandong, 2012–2017^a^.

ATC classification		2012	2013	2014	2015	2016	2017
J01A	TETRACYCLINES						
J01AA	Tetracyclines	0.03	0.02	0.19	0.18	0.13	0.13
J01C	BETA-LACTAM ANTIBACTERIALS, PENICILLINS						
J01CA	Penicillins with extended spectrum	2.30	1.96	2.71	2.93	3.01	2.41
J01CE	Beta-lactamase sensitive penicillins	2.59	1.45	1.27	0.86	0.66	0.55
J01CF	Beta-lactamase resistant penicillins	0.16	0.22	0.19	0.17	0.11	0.09
J01CR	Combinations of penicillins, incl. beta-lactamase inhibitors	0.43	0.50	0.61	1.14	1.18	1.08
J01D	OTHER BETA-LACTAM ANTIBACTERIALS						
J01DB	First-generation cephalosporins	1.40	2.05	1.72	1.77	1.20	0.90
J01DC	Second-generation cephalosporins	1.12	1.29	1.54	1.31	1.04	1.07
J01DD	Third-generation cephalosporins	2.21	2.57	2.56	2.43	1.52	1.33
J01DE	Fourth-generation cephalosporins	0.16	0.09	0.06	0.05	0.03	0.03
J01DF	Monobactams	0.09	0.02	0.03	0.03	0.02	0.02
J01DH	Carbapenems	0.03	0.03	0.04	0.05	0.06	0.08
J01E	SULFONAMIDES AND TRIMETHOPRIM						
J01EE	Combinations of sulfonamides and trimethoprim, incl. derivatives	0.04	0.05	0.00	0.00	0.00	0.00
J01F	MACROLIDES, LINCOSAMIDES, AND STREPTOGRAMINS						
J01FA	Macrolides	2.01	2.47	2.32	1.93	1.64	1.38
J01FF	Lincosamides	0.25	0.16	0.11	0.24	0.20	0.28
J01G	AMINOGLYCOSIDE ANTIBACTERIALS						
J01GA	Streptomycins	0.01	0.01	0.01	0.05	0.00	0.00
J01GB	Other aminoglycosides	0.55	0.88	0.65	0.69	0.42	0.25
J01M	QUINOLONE ANTIBACTERIALS						
J01MA	Fluoroquinolones	1.24	1.42	1.43	1.50	1.20	0.97
J01MB	Other quinolones	0.82	1.09	1.01	1.46	0.64	0.36
J01X	OTHER ANTIBACTERIALS						
J01XA	Glycopeptide antibacterials	0.01	0.01	0.01	0.01	0.01	0.01
J01XD	Imidazole derivatives	0.45	0.53	0.56	0.62	0.42	0.38
J01XE	Nitrofuran derivatives	0.12	0.15	0.02	0.00	0.00	0.00
J01XX	Other antibacterials	0.03	0.05	0.04	0.02	0.03	0.04

a Expressed in DDDs per 1,000 inhabitants and per day.

Antibiotics categorized under J01BA and J01XC are negligible and thus not listed herein.

The antibiotic sales were finally converted into DDD per 1,000 inhabitants per day (DID) at the level of the active substance. As there was no direct access to the exact number of population covered by the sample facilities, we calculated the weighted population as a proxy based on the following equation. This calculation process was under two assumptions: no significant difference existed in (1) the distribution of the sample facilities and (2) the population distribution covered by the sample facilities (Tang et al., 2018).

(1) Y=Pi*ni/Ni

*Y*: Covered inhabitants in a given year;
*Pi*: Total population in a given year;
*ni*: Number of sample facilities;
*Ni*: Number of total facilities.

We collected the relevant census data for calculating inhabitants from the Shandong Health Statistics Yearbook (Shandong Health and Family Planning Commission, 2018) and Shandong Statistics Yearbook (Shandong Provincial Bureau of Statistics, 2018).

## Results

### Total Antibiotic Use

**Figure 1:**
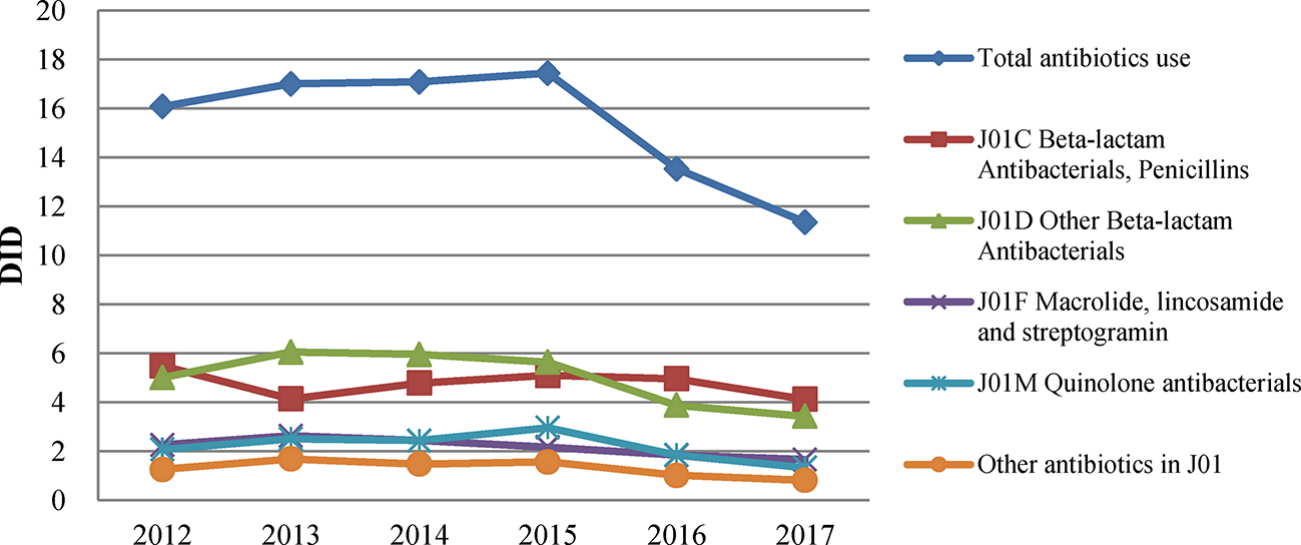
Antibiotic consumption in Shandong, China, 2012–2017.

The total consumption of antibiotics among all health care settings in Shandong increased from 16.07 DID in 2012 to a peak of 17.44 DID in 2015 and then decreased to 11.35 DID in 2017, with a 34.90% reduction (**Figure 1**). Total consumption had the sharpest drop in 2016. The proportion of antibiotics in total pharmaceutical expenditure continued to decline, from 16.23% in 2012 to 12.87% in 2017. Total pharmaceutical expenditure in Shandong increased at an average rate of 15.66% in the first 4 years and then decreased by 25.96% in 2017.

### Antibiotic Use by Class

In 2017, the most commonly used antibiotic class in Shandong was J01C (beta-lactam antimicrobials, penicillins), accounting for 36.32% of total consumption, followed by J01D (other beta-lactam antimicrobials; 30.16%), J01F (macrolides, lincosamides, and streptogramins; 14.62%) and J01M (quinolone antibacterials; 11.74%).

**Figure 2:**
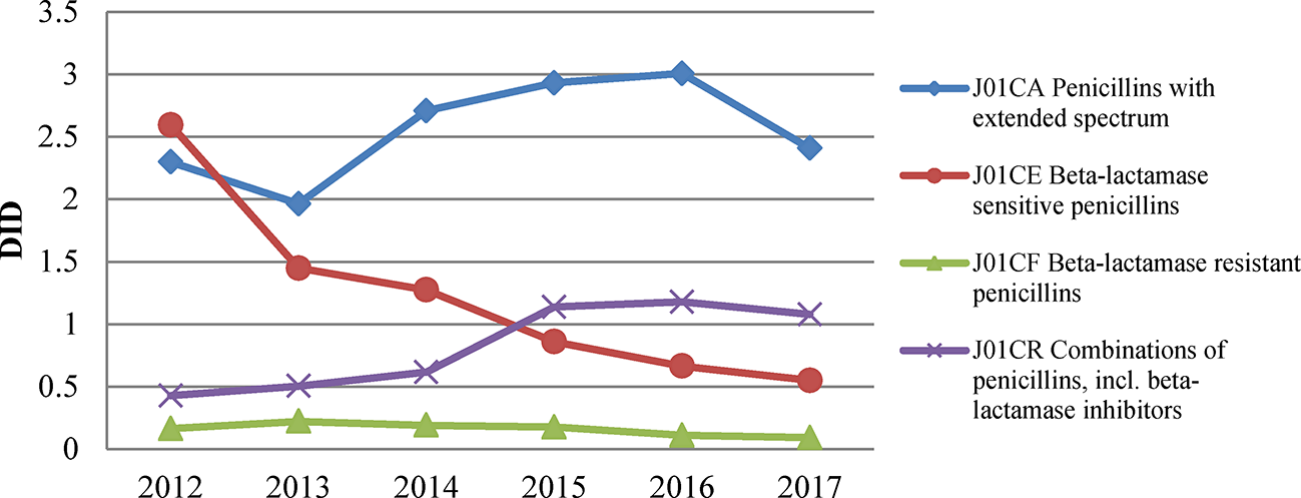
Four types of penicillin consumed in Shandong, China, 2012–2017.

During the study period, penicillin consumption (J01C) was volatile, as illustrated in **Figure 1**. It showed an obvious upward trend from 4.13 DID in 2013 to 5.10 DID in 2015, and then declined afterwards. Such a trend mainly shaped the changes in total antibiotic use over the period. Of the different types of penicillin, extended-spectrum penicillin (J01CA) was the most used, accounting for 21.21% of the total antibiotic consumption in 2017. After a sustained increase in the use of extended-spectrum penicillin from 1.96 DID in 2013 to 3.01 DID in 2016, there was a significant decline in 2017, with a decrease of about 20% (**Figure 2**). Usage of narrow-spectrum penicillin (J01CE) was reduced consistently between 2012 and 2017, from 2.59 to 0.55 DID. However, the combined use of penicillin (J01CR, mainly piperacillin and enzyme inhibitor) soared the most in all antibiotics during this period, with an increase of 0.65DID (**Figure 2**).

Overall, the use of cephalosporins (J01D) declined over the years, from a peak of 6.05 DID in 2013 to 3.42 DID in 2017 (**Figure 1**). The cephalosporin consumption was mainly in the third generations. The sales of first, second, and third generations of cephalosporin contributed to 26.29%, 31.26%, and 38.85% of the total consumption of cephalosporin, respectively. Similarly, their consumption also declined over the years (**Table 1**).

A similar downward trend was observed in J01F (macrolides, lincosamides, and streptogramins) consumption from 2.63 DID in 2013 to 1.66DID in 2017. Macrolides and lincosamides accounted for 83.13% and 16.87% of the total J01F consumption in 2017, respectively. The consumption of quinolone antibacterials (J01M) started at 2.07 DID in 2012, increased to 2.96 DID in 2015, and then decreased to 1.33 DID in 2017. Fluoroquinolones (J01MA) were the most commonly used quinolone products in Shandong, accounting for 72.93% of the total consumption of quinolones.

The consumption of carbapenems increased steadily between 2012 and 2017, from 0.029 to 0.08 DID, whereas that of glycopeptides remained stable. These two classes of antibiotics were only used in hospitals (**Table 1**).

The number of antibiotic agents consumed in Shandong decreased from 86 in 2012 to 73 in 2017. **Table 2** presents the top 10 antibiotics consumed in each year. Among them, seven antibacterial drugs, namely, benzylpenicillin, amoxicillin, ceftriaxone, cephalexin, azithromycin, levofloxacin, and cefuroxime, could be always observed. The five most used antibiotics in 2017 were amoxicillin (2.21 DID), amoxicillin and beta-lactamase inhibitor (0.78 DID), levofloxacin (0.77 DID), azithromycin (0.58 DID), and benzylpenicillin (0.55DID).

**Table 2:** Top 10 most used antibiotics in Shandong, 2012–2017.

Rank	2012		2013		2014		2015		2016		2017	
	ATC5	DID	ATC5	DID	ATC5	DID	ATC5	DID	ATC5	DID	ATC5	DID
1	J01CE01	2.59	J01CA04	1.66	J01CA04	2.34	J01CA04	2.70	J01CA04	2.79	J01CA04	2.21
2	J01CA04	2.03	J01CE01	1.45	J01CE01	1.27	J01MB04	1.46	J01MA12	0.87	J01CR02	0.78
3	J01DD04	1.12	J01DB01	1.33	J01DD04	1.23	J01MA12	1.10	J01CR02	0.78	J01MA12	0.77
4	J01MB04	0.82	J01DD04	1.31	J01DB01	1.03	J01DD04	0.92	J01FA10	0.71	J01FA10	0.58
5	J01DB01	0.81	J01MB04	1.09	J01MB04	1.01	J01CE01	0.86	J01DB01	0.67	J01CE01	0.55
6	J01FA06	0.68	J01FA06	0.95	J01MA12	1.00	J01FA10	0.73	J01CE01	0.66	J01DB01	0.50
7	J01FA10	0.66	J01MA12	0.93	J01FA06	0.78	J01DB01	0.71	J01MB04	0.64	J01DC02	0.43
8	J01MA12	0.66	J01FA10	0.67	J01DC02	0.71	J01CR02	0.61	J01DD04	0.58	J01DD04	0.41
9	J01FA09	0.51	J01FA09	0.63	J01FA09	0.65	J01DC02	0.59	J01DC04	0.42	J01DC04	0.41
10	J01DC02	0.48	J01DC02	0.60	J01FA10	0.62	J01DB04	0.58	J01DC02	0.37	J01FA06	0.37

### Antibiotic Use by Administration Route

**Figure 3:**
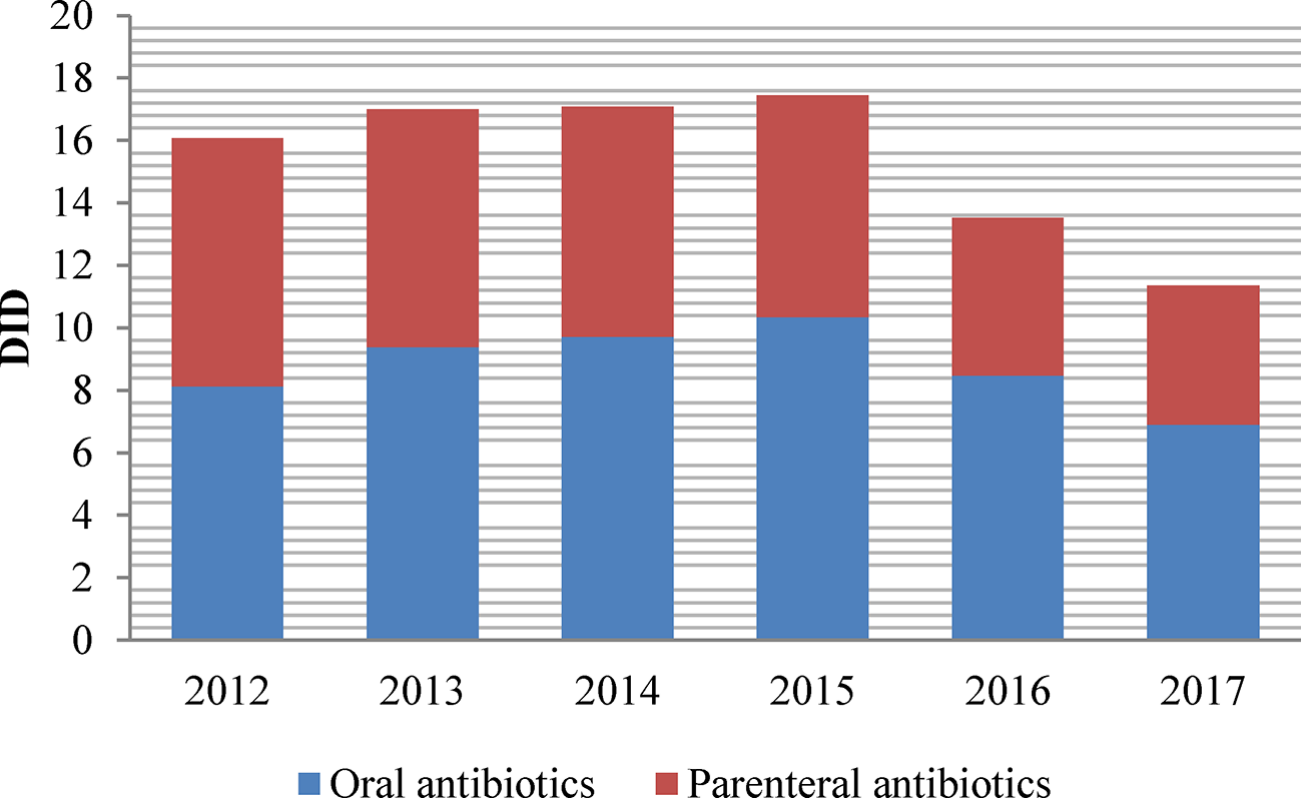
Consumption of oral and parenteral antibiotics in Shandong, China, 2012–2017.

Oral antibiotics were more prevalent over the entire study period (**Figure 3**). In 2017, the total use of parenteral antibiotics in Shandong accounted for 39.22% of the total DID, representing 89.74% of total drug expenditure. In 2017, the proportion of drugs used in parenteral format was lowest in PHCs (33.77%) and higher in secondary (47.67%) and tertiary (45%) hospitals. Parenteral antibiotics consumption continuously decreased from 7.94 DID in 2012 to 4.45 DID in 2017, by 11% per year on average. The consumption of oral preparations of antibiotics started at 8.13 DID in 2012, increased to 10.34 DID in 2015, and then decreased to 6.90 DID in 2017.

### Antibiotic Use by Type of Health Care Institution

**Figure 4:**
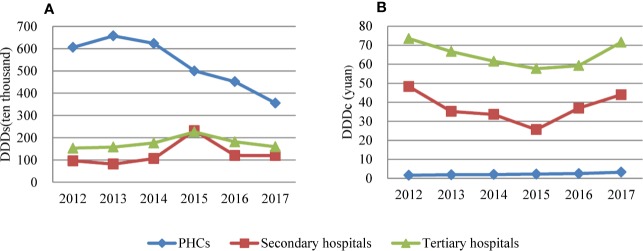
**(A)** Consumption of antibiotics and **(B)** cost of antibiotics at different levels of health care facility in Shandong, China, 2012–2017.

The pattern of antibiotic consumption in different types of health care institutions is demonstrated in **Figure 4**. In PHCs, antibiotic use showed a decreasing pattern and total consumption fell by 41% from 2012 to 2017. In hospitals, antibiotic consumption began to decline after reaching its peak in 2015. Both secondary and tertiary hospitals showed a similar varying trend. Compared with 2012, the 2017 consumption showed a small increase in hospital, but then it was compensated by a decrease in community care. The average daily cost of antibiotics in hospitals was much higher than that in PHCs, although the daily cost of antibiotics in PHCs increased yearly. In 2017, the average daily cost of antibiotics was CNY 3.28 in PHCs, CNY 43.98 in secondary hospitals, and CNY 71.53 in tertiary hospitals.

## Discussion

Our study quantified the antibiotic consumption in China in a six-year span, which would be informing for decision-makers, health care providers, as well as the public.

The results of this study showed that Shandong province reported a lower antibiotic consumption in China in 2017, compared with the global average (15.7 in 2015) (Klein et al., 2018). Over the study period, the percentage of total pharmaceutical expenditures on antibiotics steadily dropped and total antibiotic consumption presented a substantial decreasing trend after 2015. These changes coincided with the multiple interventions that regulate the use of antibiotics in China, including the 2012 Administrative Rules for the Clinical Use of Antibiotics issued by the Ministry of Health. This administration intervention appeared to work better according to the findings of this study and others (Zhang et al., 2019). In addition to persuasive guidelines, penalties for violating these administrative rules and measures, such as downgraded accreditation and service fee levels and dismissal of managers, were applied. Medical workers involved may lose permission to prescribe antibiotics, or even have their medical registration revoked.

In addition, a comprehensive reform of public hospitals has been carried out nationwide since 2015 (General Office of the State Council, 2015). The core content of this reform was to expand the zero mark-up drug policy to secondary and tertiary public hospitals, with the goal of completely eliminating the harmful incentives for drug abuse. Previous studies have indicated that the zero mark-up drug policy could help reduce the prescription rate of antibiotics (Wei et al., 2017). Therefore, this reform may have interacted with the antimicrobial stewardship programs and played an important role in improving antibiotic use. Public hospitals in Shandong province contribute to more than 85% of the total number of patients admitted to all medical institutions and more than 90% of the total health expenditure (Shandong Health and Family Planning Commission, 2018). The mixed effects of these two interventions could be an explanation for the dramatic decline in antibiotic consumption in 2015, as shown in this study.

Penicillin was found to be the most consumed antibiotic in this study. This is in line with the results found in Europe and the United States (Centers for Disease Control and Prevention, 2015; European Centre for Disease Prevention and Control, 2016). However, the findings of the present study regarding major antibiotic use were different from previous studies based on Chinese tertiary hospitals. This may be related to restrictions on the use of antibiotics by different levels of health care institutions (Wushouer et al., 2017; Qu et al., 2018). In addition, the widespread use of broad-spectrum antibiotics in China is noteworthy. In Shandong, the consumption of penicillin with extended spectrum was much higher than that of other subgroups. The combined use of penicillin, commonly defined as extended-spectrum antibiotics, showed an upward pattern and increased the most over 6 years. The third-generation cephalosporins, which has a more extended-spectrum, accounted for more than half of the total cephalosporin consumption. A distinct advantage of broad-spectrum antibiotics is that they require minimal recognition of infectious pathogens prior to treatment compared with narrow-spectrum antibiotics. However, the patient’s normal flora will be affected by them, and antibiotic resistance might be accelerated (Kaye et al., 2001). Therefore, necessary measures should be taken to further standardize the prescription of antibiotics, targeting at avoiding the abuse of broad-spectrum antibiotics.

The most used antibiotic agent was amoxicillin, followed by amoxicillin and beta-lactamase inhibitor, levofloxacin, azithromycin, and benzylpenicillin. These results are comparable to those of a study conducted in 48 primary health care facilities sampled from six provinces in China (Wang et al., 2014) and consistent with those from other Asian countries, such as Malaysia and India (Sharma et al., 2012; Shamsuddin et al., 2016).

Findings in this study showed a declining antibiotic use. This may be partly attributed to the fact that antibiotics are forced into three categories based on doctors’ prescription rights (unrestricted, restricted, and specialist use only), which was part of the antibiotic stewardship campaign in 2012 (Wushouer et al., 2017). This measure made antibiotics more difficult to be prescribed. A similar decline in antibiotic use in China can also be observed in other studies (Bao et al., 2015; Wushouer et al., 2017).

The use of carbapenems and glycopeptides, which are often regarded as the last-line treatment for multidrug-resistant bacteria, remains at a very low level in Shandong, because their use is highly restricted and requires pre-authorization. Notably, however, carbapenems consumption more than doubled during the study period. The surveys in other regions in China had a similar finding and indicated that imipenem, meropenem, and biapenem were the mainly used agents (Xu and Jia, 2015). Its increase may be a response to the rising prevalence of MRSA and ESBL-producing bacteria, which were identified in epidemiological surveillance studies (Xiao et al., 2013).

Several studies have indicated a very high level of antibiotics consumption in primary health care facilities in China (Wang et al., 2014; Sun et al., 2015). In this study, the consumption in PHCs displayed a declining trend over six years, indicating a positive effect of a series of restrictive measures, which have been imposed in national essential medicines policies since 2009, on antibiotic prescribing practices. These measures include limiting the availability of antibiotics and setting a zero-markup for sales of medicines in primary care. Comparatively, the obvious reduction of antibiotic consumption in hospitals was observed after 2015. As discussed above, the downward trend may be caused by the zero mark-up policy implemented in secondary and tertiary hospitals in Shandong. Moreover, the cost of antibiotics used in hospitals was much higher than that in PHCs; this could be a key to medical cost control.

Although there has been a decline of 3.49 DID since 2012, the parenteral antibiotics presented nearly 40% of total consumption and more than 80% of total expenditure in 2017. The high prevalence of antibiotics and injections may be related to the inertia in a physician’s practice and high demands of patients (Yang et al., 2014). In many cases, patients prefer antibiotics and injections for rapid recovery; therefore, antibiotics are often prescribed in combination with injections (Li, 2014). Health education and interventions are supposed to be set up for the public to promote the proper use of antibiotics (Ye et al., 2017).

The importance of this study lies providing up-to-date evidence on antibiotics consumption in China by different levels of health care facilities in Shandong province. Valuable information could be provided to national and provincial governments for developing more clearly targeted antibiotic stewardship policies. The limitations of this study are as follows. First, the antibiotic consumption data were only collected from 39 public health care institutions. Total consumption of antibiotics in Shandong province might be under-estimated. However, the trends and patterns of antibiotic use can be correctly reflected. Second, the population denominator used in this study did not consider patient flow between regions; this study may have underestimated antibiotic consumption. Finally, this study analyzed antibiotic sales data, which do not directly reflect the actual use of medicines and types of infections requiring antibiotics. The appropriateness of antibiotic prescribing practices was also not evaluated. Although the method used is internationally accepted, the resulting DDD and DID data cannot be followed back to the demand of the individual patient. In the future, prescription analysis and AMR monitoring must be combined to assess antibiotic consumption based on population, which can inform better the decision-making and improve clinical significance.

## Conclusion

This study revealed a substantial decline in antibiotic consumption in China in 2012-2017, and a consistent preference for penicillins, cephalosporins, macrolides, and fluoroquinolones. The large consumption of broad-spectrum antimicrobials, high proportion of parenteral antibiotic use, and increased use of last-resort antibiotics attracted public health concerns. Using antibiotics in secondary and tertiary hospitals may be the key to controlling medical costs. Continued efforts are needed to obtain more evidence to inform policies and thereby optimize antibiotic prescribing and minimize antibiotic resistance.

## Data Availability Statement

All datasets generated for this study are included in the article/supplementary material.

## Author Contributions

YS and TZ contributed to the conception and design of the study. YS organized the database. YS, ZH, and KS conducted the statistical analysis. YS finished the first draft of the manuscript. All authors made contributions to manuscript revision and read and approved the submitted version.

## Funding

This study was funded by the National Natural Science Foundation of China (grant number 71503149), the Innovation Project of Shandong Academy of Medical Sciences, and Academic promotion programme of Shandong First Medical University. The funders had no role in study design, data collection and analysis, decision to publish, or preparation of the manuscript.

## Conflict of Interest

The authors declare that the research was conducted in the absence of any commercial or financial relationships that could be construed as a potential conflict of interest.
